# A power allocation strategy for fuel cell ship considering fuel cell performance difference

**DOI:** 10.1038/s41598-023-37076-2

**Published:** 2023-06-19

**Authors:** Wei Cao, Pan Geng, Xiaoyan Xu, Yi Guo, Zhanxin Ma

**Affiliations:** 1 Logistics Engineering College, Shanghai Maritime University, Shanghai, 201306 China; 2 China Institute of FTZ Supply Chain, Shanghai, China

**Keywords:** Engineering, Electrical and electronic engineering

## Abstract

This paper focuses on designing a power allocation strategy for a fuel cell ship. The performance of the fuel cell varies during operation, so a power allocation strategy considering fuel cell performance differences is proposed, which consists of two layers. In the first layer, the maximum power and maximum efficiency of each fuel cell system (FCS) are updated in real-time with an online parameter identification model, which is composed of the fuel cell semi-empirical model and adaptive Kalman filter. The second layer takes the state of charge of the battery energy storage system, the maximum power, and the maximum efficiency as inputs for power allocation. Compared with the equal allocation strategy and daisy chain strategy, the total hydrogen consumption reduces by 5.3% and 15.1% and the total output power of the FCS with poor performance reduces by 14.1% and 15.7%. The results show that the proposed method can improve the efficiency of the ship power system and reduce the operational burden of the FCS with poor performance.

## Introduction

The data show that international shipping accounted for 2.89% of global anthropogenic greenhouse gas emissions in 2018, and without stringent regulations, this percentage will rise. To reduce CO_2_ from the ship, the International Maritime Organization (IMO) has developed regulations^1^. In consequence, ship manufacturers are working to incorporate fuel cells and renewable energy into ship power systems due to their high efficiency and pollution-free characteristics^2,3^. The FCS suffers from a slow dynamic response and requires a couple with ESS for the application. The efficiency of a hybrid energy system (HES) mostly relies on the power allocation strategy^4^.

The power allocation strategy can be divided into rule-based and optimization-based. The rule-based methods include support vector machines, frequency control, and fuzzy control. In^5^, to improve the dynamic performance of the HES and extend the lifetime of the fuel cell, a power allocation strategy based on support vector machines and frequency control is suggested for the fuel cell ship. In^6^, a fuzzy control-based power allocation strategy is proposed to achieve a reasonable distribution of load power between Li-ion batteries and supercapacitors. Although rule-based approaches have a low computational burden, it is difficult to obtain a globally optimal solution. Therefore, intelligent algorithms and equivalent energy minimization strategy (ECMS) are used to design optimization-based power allocation strategies. For example, a power allocation strategy based on a sine cosine algorithm is proposed for a ferry boat^7^. The result shows the HES based on fuel cell and the battery has a higher performance. Hasanvand et al*.*^8^ used deep reinforcement learning to address the problem of energy management. The proposed method is validated with a real load profile. In^9^, an adaptive model predictive control is proposed for an all-electric ship and the cost function including power compensation error and energy storage system loss is established.

These methods are based on the premise of constant fuel cell parameters. However, the performance of fuel cells can be affected by external conditions, such as temperature and pressure^10^. Therefore, it is necessary to consider the fuel cell performance when designing the power allocation strategy. For instance, the Kalman filter (KF) is used to extract the maximum power (MP) and maximum efficiency (ME) of the FCS and an adaptive power allocation strategy for the multi-stack fuel cells is proposed in^11^. In^12,13^, the recursive least squares-based parameter identification method is used to update the optimal efficiency range of the fuel cell system, and an ECMS-based power allocation strategy is proposed. In^14^, the authors develop a fuel cell voltage degradation model. To maintain consistent fuel cell performance, a droop control-based power allocation strategy is designed. Similarly, the performance of the fuel cell is also evaluated with voltage in^15^, and the characteristic curves of the fuel cell system are given for different health conditions. To improve the system economy, an adaptive energy management strategy is proposed. Moreover, In^16^, the Lyapunov-based adaptive law and the KF are used to estimate the remaining lifetime of the fuel cell. The results show that Lyapunov-based adaptive law is more accurate than KF. However, the authors do not study the power allocation strategy further.

Both the performance of the fuel cell and the power allocation strategy is critical for hybrid energy system according to the previous analysis. However, most of the power allocation strategies focus on reducing fuel consumption and do not pay attention to the inconsistencies in performance between fuel cells. The fuel cell with poor performance will degrade further if the output power of all fuel cells keeps consistent, which will affect the normal operation of the whole system^17^.

In response to the deficiencies of the previous studies, a two-layer control-based power allocation strategy is proposed in this paper, which takes into account the performance differences among fuel cells.

The main contributions are given below. First, the performance of three parameter identification methods, KF, adaptive Kalman filter (AKF), and recursive least squares (RLS), is analyzed. An online parameter identification model based on the adaptive Kalman filter is proposed to update the fuel cell semi-empirical model parameters in real-time. Second, the MP of the FCS is used to evaluate the performance of the fuel cell. To reduce the operational burden of the FCS with poor performance, a power allocation strategy considering fuel cell performance difference is proposed. For verifying the effectiveness of the method, a detailed comparison is made between the equal allocation strategy and the daisy chain strategy.

This article is organized as follows. Section II describes the ship power system model. Section III outlines the fuel cell semi-empirical model and the AKF. The results of online parameter identification are analyzed. In section IV, a two-layer power allocation strategy is proposed for the fuel cell ship. In section V, the simulation result is analyzed. Finally, the conclusions are presented in section VI.

## Ship power system

**Figure 1:**
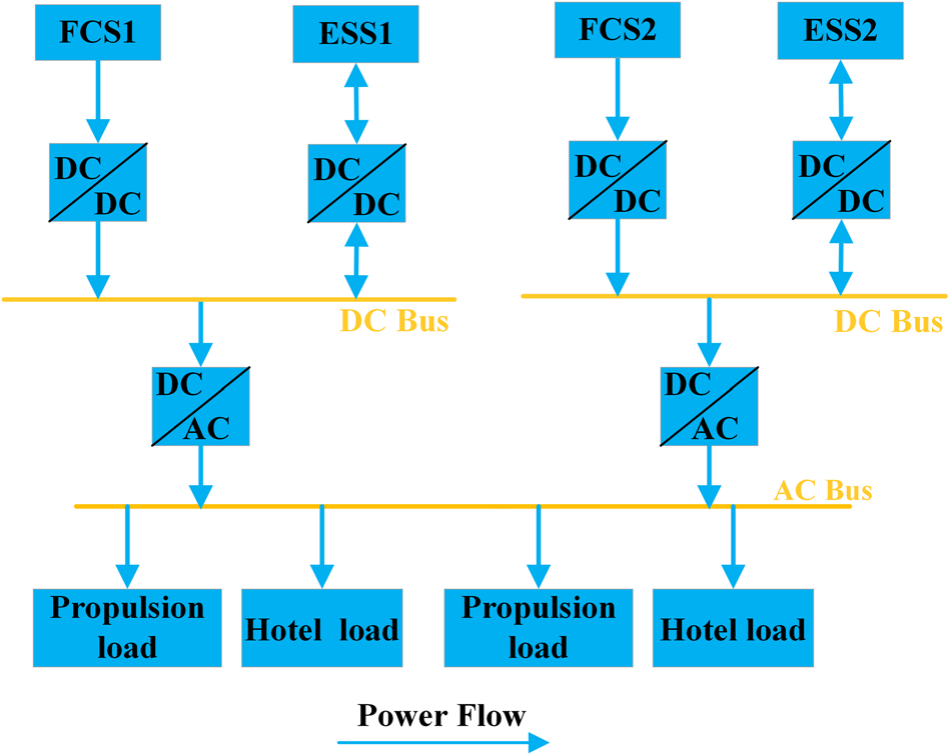
Ship power system model.

The ship power system model is shown in Fig. 1, which includes two FCSs and two ESSs. The FCS is the primary power source, and the ESS is the auxiliary power source. Due to the slow dynamic performance of FCS, the ESS is used to follow the fast load variations.

### Fuel cell system

**Figure 2:**
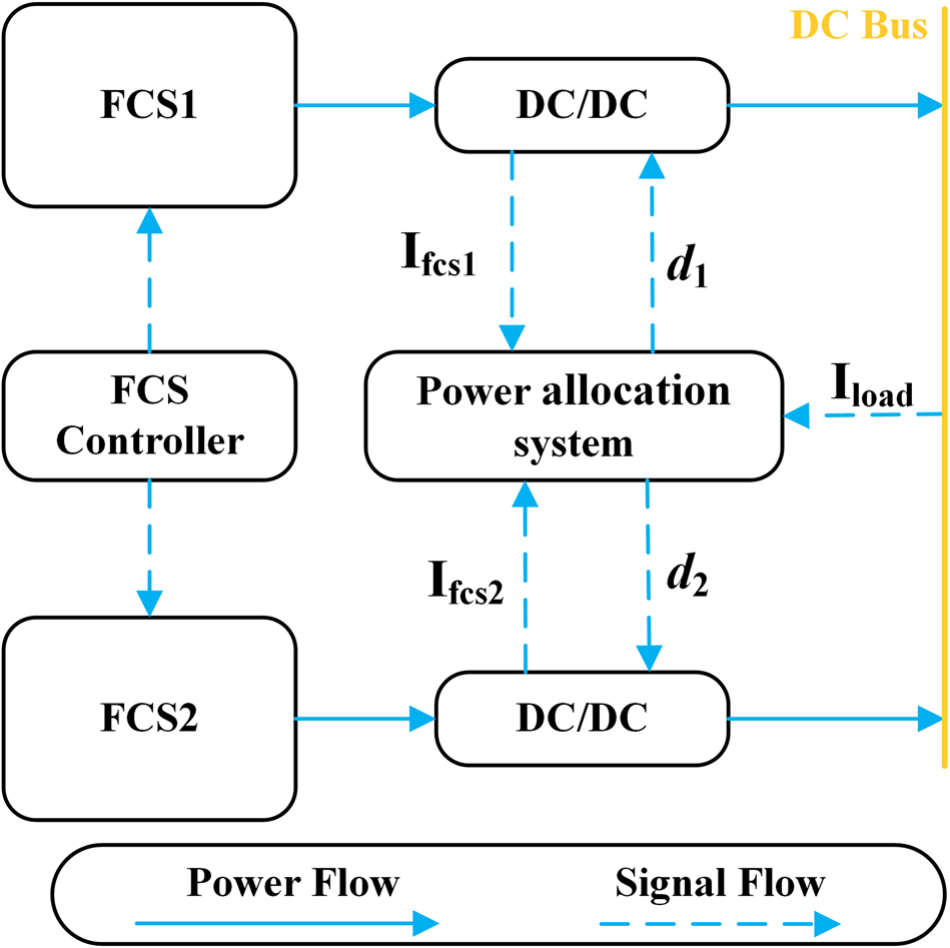
The topology of FCS.

The performance of the multi-stack FCS depends on its topology. The ship power system cannot operate normally if a single FCS fails with serial-connected architecture. However, the FCS is independently controlled by the DC-DC converter with the parallel-connected architecture, which can enable fault isolation, and the durability and efficiency of the system are better than the former one^14^. This paper adopts the parallel-connected architecture, as shown in Fig. 2. The output power of the FCS1 and FCS2 is controlled by adjusting the duty cycle *d*_1_ and *d*_2_ of the DC-DC converter.

### Battery energy storage system

**Figure 3:**
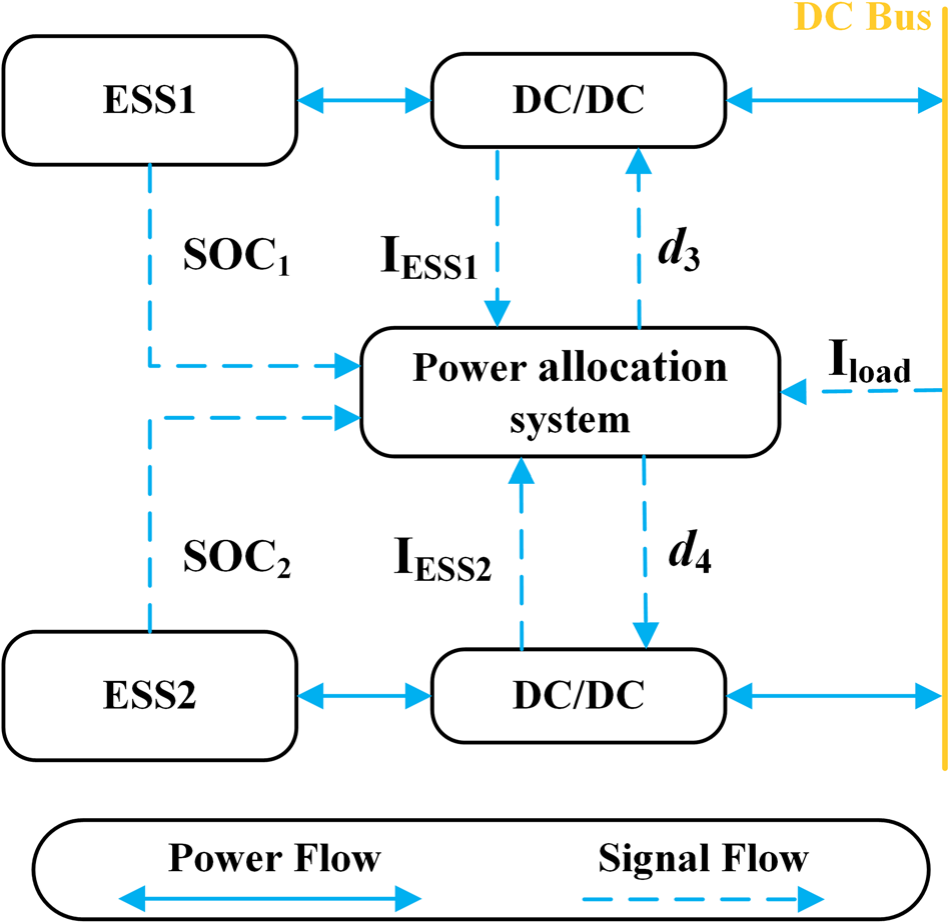
The topology of ESS.

The parallel-connected architecture is adopted for ESS, as shown in Fig. 3. Since the ESS has both charging and discharging states, it is connected to the DC bus via a bidirectional DC-DC converter. The output power of the ESS1 and ESS2 is controlled by adjusting the duty cycle *d*_3_ and *d*_4_ of the DC-DC converter.

## Online parameter identification

**Figure 4:**
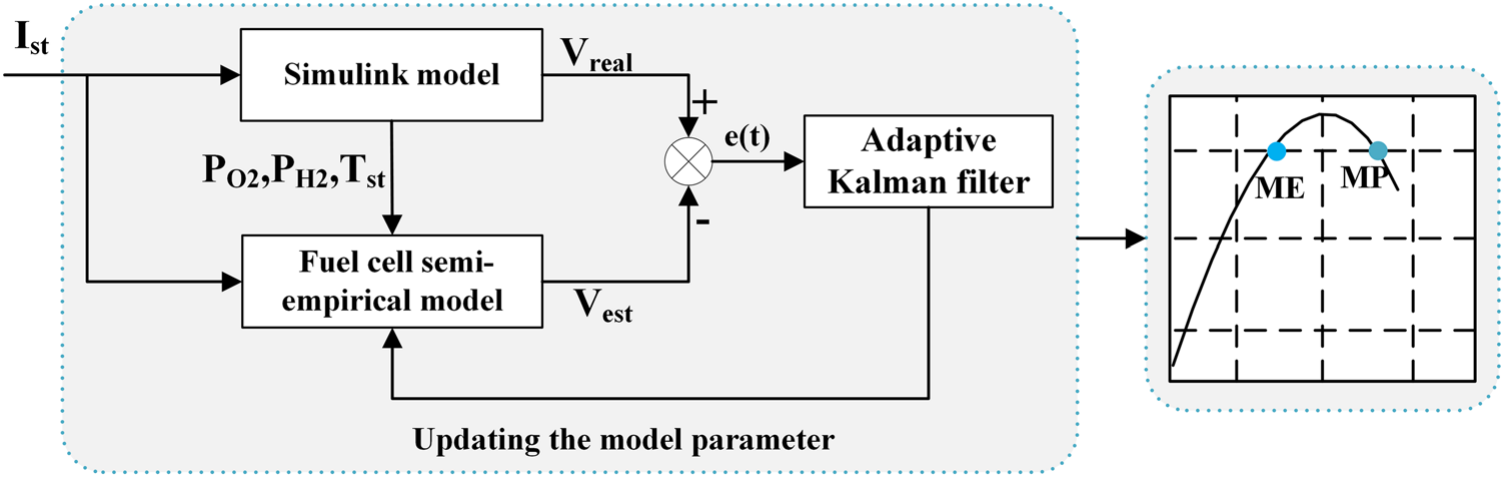
Online parameter identification.

The process of online parameter identification is shown in Fig. 4. The parameters of the fuel cell semi-empirical model are updated by AKF, and the characteristic curves of FCS are extracted with the updated model. The ME and MP of the FCS can be obtained from these curves.

### Fuel cell semi-empirical model

The fuel cell semi-empirical model proposed by Amphlett is adopted in this paper to estimate the voltage of the fuel cell and its general formulation given as (1)–(5)^18^. According to the literature^19^, this model is more accurate than others. The fuel cell output voltage is calculated by Eq.(1).

1 $$V_{st} = N_{cell} * \left( {E_{Nernst} + V_{act} + V_{ohmic} + V_{con} } \right)$$

where *N*_*cell*_ is the number of cells; *E*_*Nernst*_ is the reversible cell potential; *V*_*act*_ is the activation loss; *V*_*ohmic*_ is the ohmic loss; *V*_*con*_ is the concentration loss. The *E*_*Nernst*_ is determined by the stack temperature, and the pressure on the anode side and the cathode side, as shown in Eq. (2).

2 $$\begin{aligned} E_{Nernst} = & 1.229 - 0.85 \times 10^{ - 3} \times (T_{st} - 298.15) + 4.3085 \times 10^{ - 5} \\ {\kern 1pt} & \times T_{st} \times \left[ {\ln \left( {P_{{H_{2} }} } \right) + \ln \left( {\sqrt {P_{{O_{2} }} } } \right)} \right] \\ \end{aligned}$$

where *T*_*st*_ is the stack temperature; *P*_*H2*_ is the hydrogen partial pressure on the anode side; *P*_*O2*_ is the oxygen partial pressure on the cathode side. The activation voltage loss is mostly due to catalyst layer aging, as shown in Eq. (3).

3 $$\left\{ \begin{aligned} V_{act} = & \xi_{1} + \xi_{2} T_{st} + \xi_{3} T_{st} \ln \left( {CO_{2} } \right) + \xi_{4} T_{st} \ln \left( {i_{st} } \right) \\ CO_{2} = & P_{{O_{2} }} /\left( {5.08 \times 10^{6} \times \exp \left( { - \frac{498}{{T_{st} }}} \right)} \right) \\ \end{aligned} \right.$$

where *i*_st_ is the stack current; *ξ*_n_(n = 1, 2, 3, 4) is the semi-empirical parameter. The ohmic voltage loss is attributed to the proton exchange membrane aging and can be written as Eq. (4).

4 $$V_{ohmic} = - i_{st} \times \left( {\zeta_{1} + \zeta_{2} \times T_{st} + \zeta_{3} \times i_{st} } \right)$$

where *ζ*_m_(m = 1, 2, 3) is the semi-empirical parameter. The concentration voltage loss is caused by the reduction of the maximum current density. As shown in Eq. (5).

5 $$V_{con} = B\ln \left( {1 - \frac{{i_{st} }}{{i_{\max } }}} \right)$$

where *i*_*max*_ is the maximum stack current; *B* is the semi-empirical parameter.

The FCS efficiency and hydrogen consumption are calculated according to the Eqs. (6)-(8)^13^.

6 $$\begin{gathered} P_{st} = V_{st} \times i_{st} \hfill \\ P_{fcs} = P_{st} - P_{aux} \hfill \\ \end{gathered}$$

7 $$\eta_{fcs} = \frac{{V_{st} }}{{N_{cell} \times 1.254}} \times \left( {1 - \frac{{P_{aux} }}{{P_{st} }}} \right)$$

8 $$m_{{H_{2} }} = \frac{{P_{fcs} }}{{\eta_{fcs} \times LHV}}$$

In these equations *P*_*aux*_ represents the auxiliary system power; *P*_*st*_ is the fuel cell stack output power; *P*_*fcs*_ is the FCS output power; *LHV* is the hydrogen lower heat value.

### Adaptive Kalman filter

The KF is essentially a recursive form of a state-optimal estimation algorithm, which is widely used in linear systems due to its simplicity and high accuracy^20–22^. The noise covariance matrices *Q* and *R* must be known in the conventional KF. However, *Q* and *R* are uncertain during the operation of the system. Therefore, the AKF is employed in this paper. The structure of AKF is as follows:

9 $$\begin{gathered} x_{k} = Ax_{k - 1} + w_{k - 1} \hfill \\ Z_{k} = Hx_{k} + \upsilon_{k} \hfill \\ \end{gathered}$$

10 $$\hat{x}_{k}^{ - } = A\hat{x}_{k - 1}$$

11 $$P_{k}^{ - } = AP_{k - 1} A^{T} + Q_{k - 1}$$

12 $$K_{k} = \frac{{P_{k}^{ - } H_{k}^{T} }}{{H_{k} P_{k}^{ - } H_{k}^{T} + R_{k} }}$$

13 $$\hat{x}_{k} = \hat{x}_{k}^{ - } + K_{k} (Z_{k} - H_{k} \hat{x}_{k}^{ - } )$$

14 $$P_{k} = \left( {I_{8 \times 8} - K_{k} H_{k} } \right)P_{k}^{ - }$$

where *x* is the state vector; A is the transition matrix, A = I_8×8_; *H* is the measurement matrix; *ω* is the system process noise vector; *υ* is the measurement noise vector;* Z* is the output; ${\widehat{x}}^{-}$ is the prior estimation of the state vector; *P* is the error covariance matrices; *R* is the measurement noise covariance matrices; *Q* is the system process noise covariance matrices; *K* is the Kalman gain.

The residuals *e*_*k*_ and the covariance of the residuals are shown in Eqs. (15)–(16) ^23^. The estimation of *C*_*k*_ is shown in Eq. (17).

15 $$e_{k} = Z_{k} - H_{k} \hat{x}_{k}^{ - }$$

16 $$E\left( {e_{k} e_{k}^{T} } \right) = C_{k} = H_{k} P_{k}^{ - } H_{k}^{T} + R_{k}$$

17 $$\hat{C}_{k} = \frac{1}{N}\sum\limits_{m = k - N + 1}^{k} {e_{m} e_{m}^{T} }$$

where *k* is the current moment; *N* is the moving window size.

The measurement data and prior estimate results are used to correct *Q* and *R* online, as shown in Eqs. (18)–(19). Assume that *Q, R,* and* P* are positive definite matrices.

18 $$\hat{Q}_{k} = K_{k} \hat{C}_{k} K_{{_{k} }}^{T}$$

19 $$\hat{R}_{k} = \hat{C}_{k} - \frac{1}{N}\sum\limits_{m = k - N + 1}^{k} {H_{m} P_{m}^{ - } H_{m}^{T} }$$

**Figure 5:**
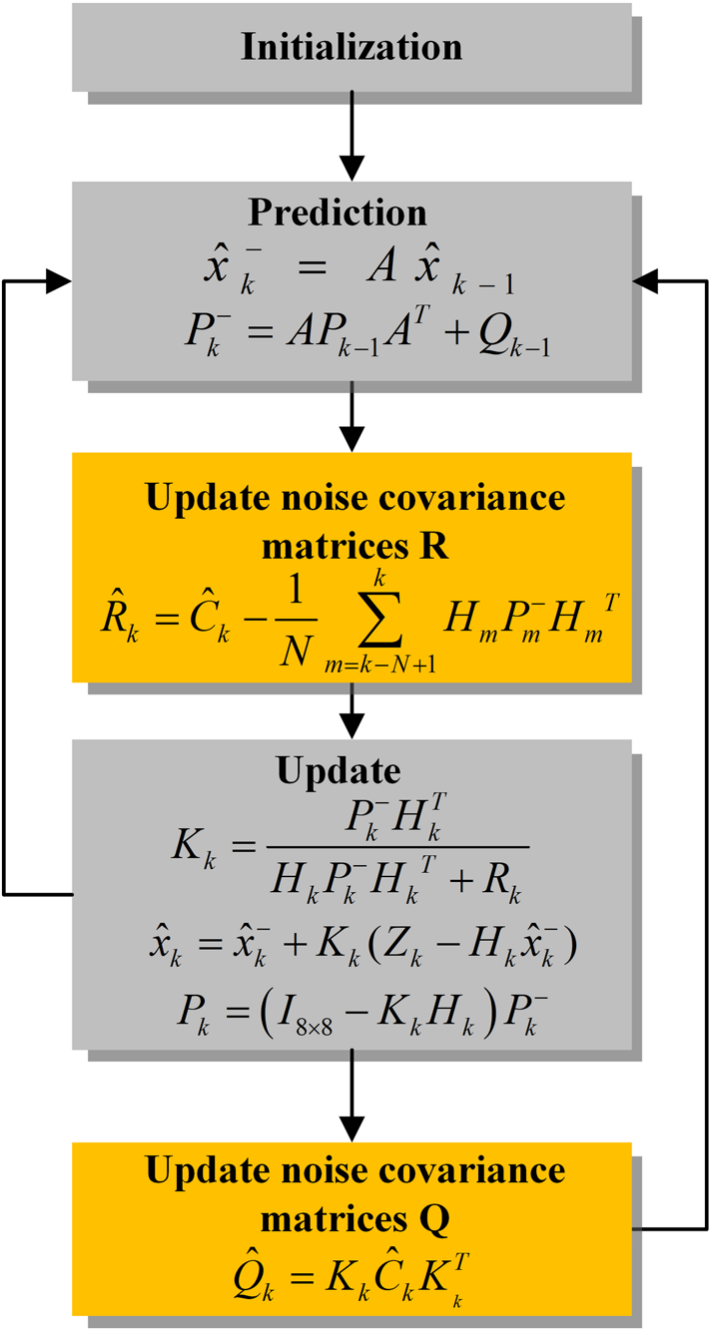
The flowchart of AKF.

The state vector consists of semi-empirical parameters, as shown in Eq. (20), and these parameters are updated by the AKF. The measurement matrix is shown in Eq. (21). The flowchart of the AKF is shown in Fig. 5.

20 $$x = \left[ {\xi_{1} ,\xi_{2} ,\xi_{3} ,\xi_{4} ,\zeta_{1} ,\zeta_{2} ,\zeta_{3} ,B} \right]^{T}$$

21 $$H = \left[ {1,T_{st} ,T_{st} \ln \left( {CO_{2} } \right),T_{st} \ln \left( {i_{st} } \right), - i_{st} , - i_{st} T_{st} , - i_{st}^{2} ,\ln (1 - \frac{{i_{st} }}{{i_{\max } }})} \right]$$

### Result analysis

**Figure 6:**
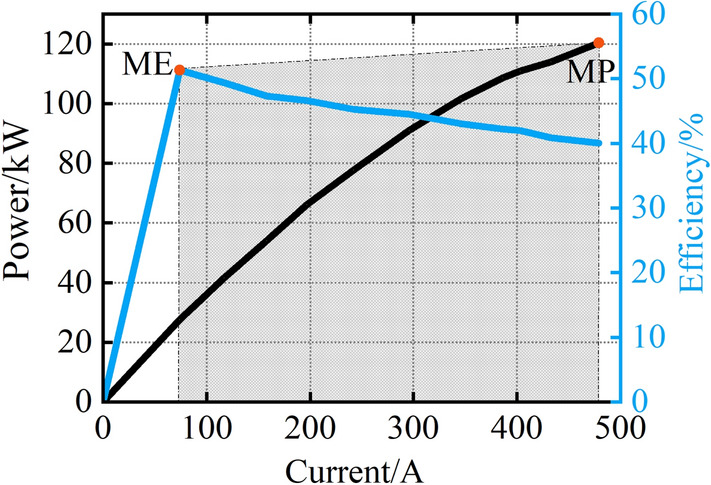
Characteristic curves.

The proton exchange membrane fuel cell (PEMFC) is used in this paper. The FCS consists of a stack module, hydrogen supply system, oxygen supply system, cooling system, etc. The characteristic curves of FCS are shown in Fig. 6. These curves are used as a reference to check the performance of different parameter identification methods. The experiment parameter is shown in Table 1. The MP and ME of the FCS are 120 kW and 51.34% respectively. The maximum stack temperature is set to 80 °C. The stack temperature is regulated by the cooling system.

**Table 1:** Experiment parameter.

Parameter	Value
Number of cells(*N*_*cell*_)	554
The maximum current(*i*_*max*_)	500A
The maximum stack temperature	80 °C
The maximum power(MP)	120 kW
The maximum efficiency(ME)	51.34%
lower heat value(*LHV*)	120 MJ/kg
Moving window size(*N*)	3

**Figure 7:**
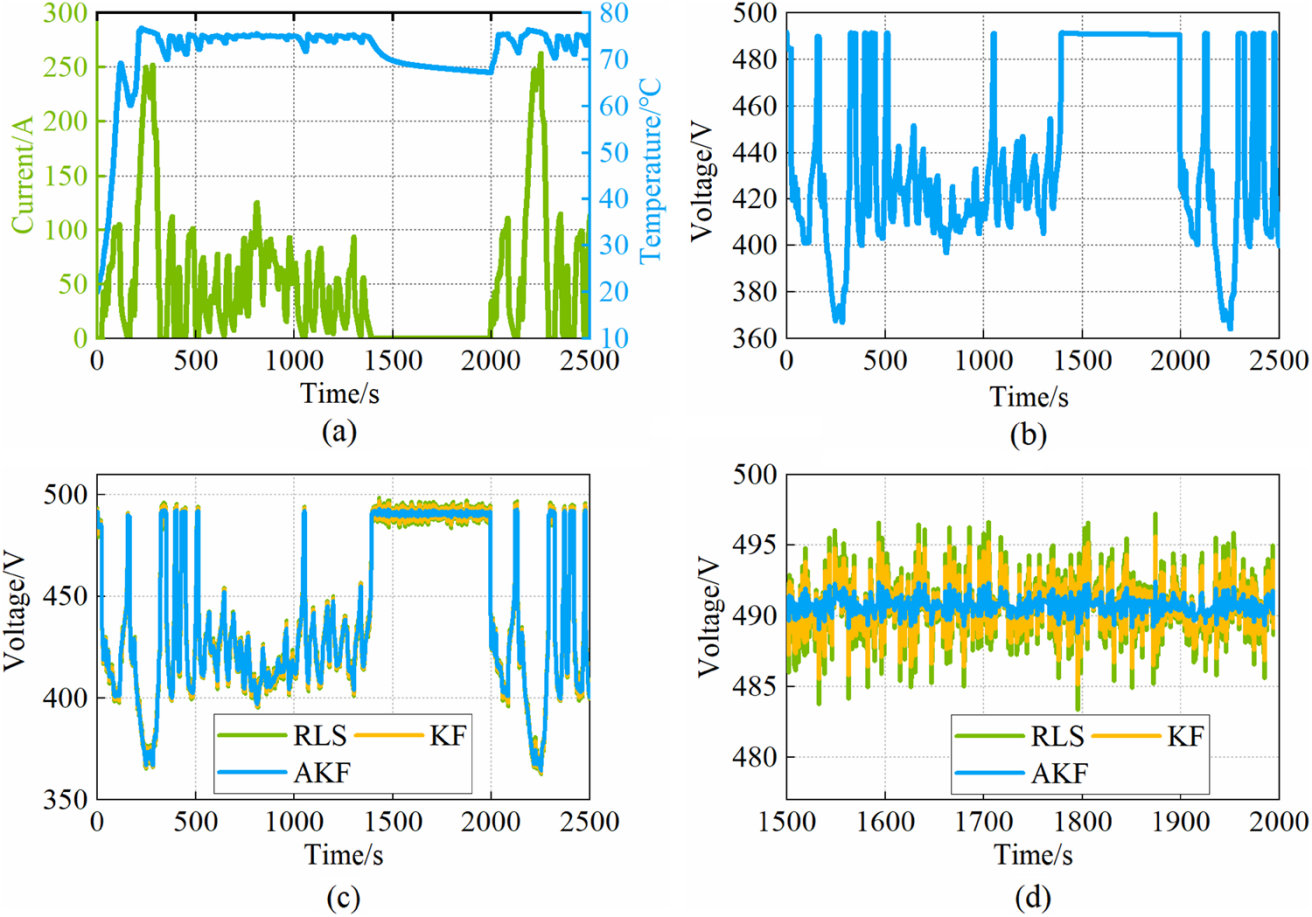
(**a**) Current and temperature curves; (**b**) The real voltage; (**c**) Voltage estimation by RLS、KF and AKF; (**d**) Voltage estimation within 1500–2000s.

The input current profile and the recorded temperature and output voltage are shown in Fig. 7a and b. The estimated result of the three methods is shown in Fig. 7c and d, the estimated voltage curve with AKF is smoother than KF and RLS.

Furthermore, the performance of AKF, KF, and RLS is compared with the root mean square error (RMSE) and mean absolute error (MAE), as shown in Eqs. (22)–(23).

22 $$RMSE = \sqrt {\frac{1}{n}\sum\limits_{i = 1}^{n} {\left( {\hat{V}_{i} - V_{i} } \right)^{2} } }$$

23 $$MAE = \frac{1}{n}\sum\limits_{i = 1}^{n} {\left| {\hat{V}_{i} - V_{i} } \right|}$$

where $\widehat{V}$ is the estimated voltage; *V* is the real voltage.

The RMSE and MAE are shown in Table 2. The RMSE and MAE with AKF are 0.7035 and 0.5631 respectively, compared with the KF and RLS, the RMSE reduces by 64% and 73.2%, and the MAE reduces by 64.6% and 73.27%.

**Table 2:** RMSE and MAE.

	AKF	KF	RLS
RMSE	0.7035	1.9898	2.6323
MAE	0.5631	1.5927	2.1069

**Figure 8:**
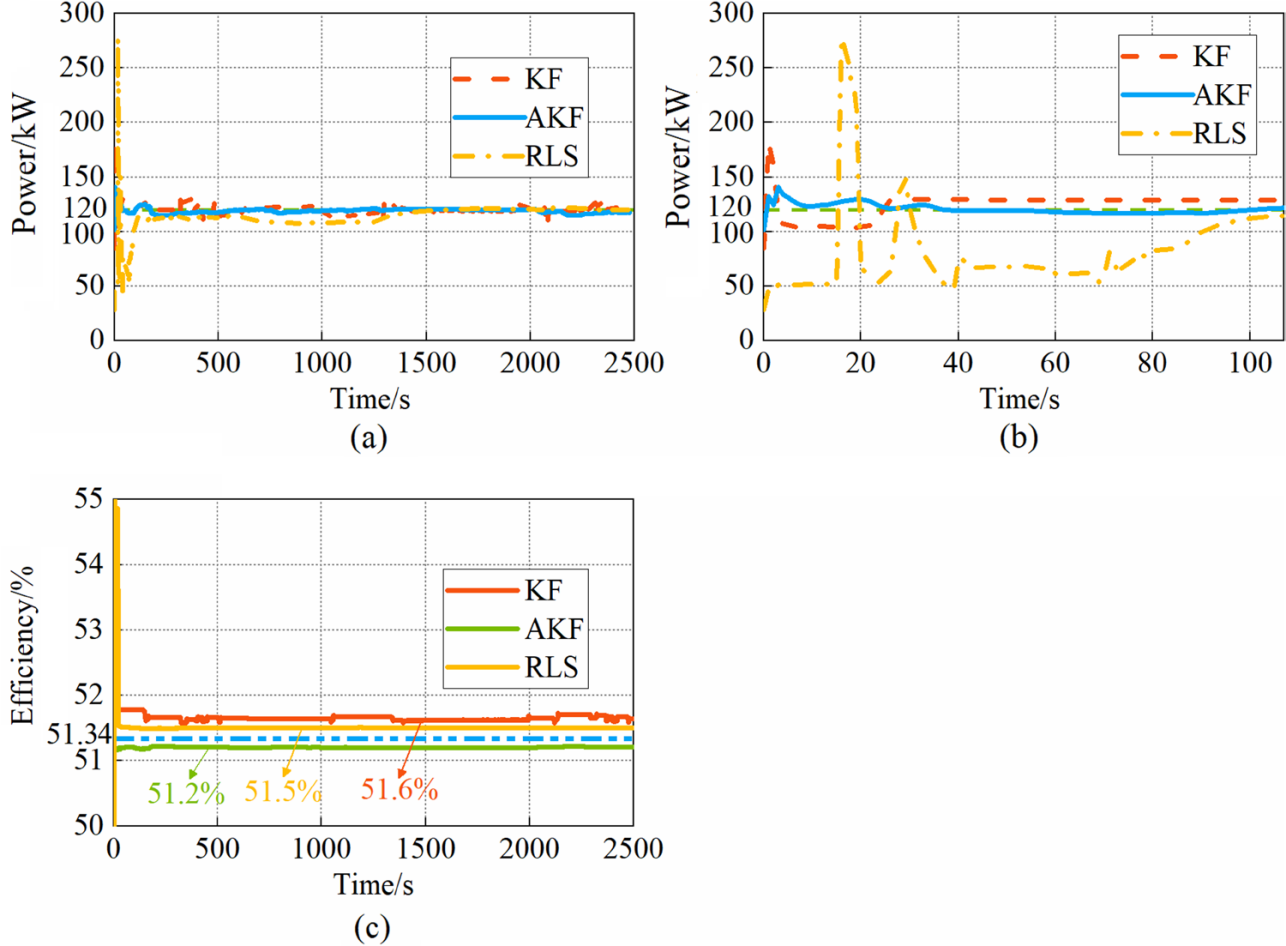
(**a**)MP estimation by KF, AKF, and RLS; (**b**) MP estimation within 0–107 s; (**c**) ME estimation by KF, AKF, and RLS.

To extract the MP and ME of the FCS, the power and efficiency curves are estimated at each simulation step. The estimated MP and ME of the FCS with the three methods are shown in Fig. 8. From Fig. 8a, all three methods can accurately estimate the MP of the FCS. From Fig. 8b, there are fluctuations in all three methods in the initial stage, however, the voltage estimation with AKF experiences fewer fluctuations compared to the KF and RLS. The AKF reaches a steady state after 14 s, however, the KF and RLS need 33 s and 107 s respectively. From Fig. 8c, the estimated ME with AKF is 51.2%, which is closer to the real value than KF and RLS. Due to the short simulation time, the estimated MP and ME of the FCS do not change.

## Power allocation strategy

A power allocation strategy considering fuel cell performance differences is proposed in this paper and compared with the equal allocation strategy and daisy chain strategy.

### Strategy 1: equal allocation strategy

The equal allocation strategy takes the load power and the state of charge (SOC) of the battery SOC as inputs and shares the load power equally among the FCSs^24^. The FCS output power is shown in Eq. (24).

24 $$P_{fcsi} \left( t \right) = P_{load} \left( t \right)/N_{fcs}$$

where *P*_*load*_ is the load power, *N*_*fcs*_ is the number of FCS.

To increase the redundancy of the ship power system, the ESS2 is used as a backup power source. In case of a fault of other power sources or overload, the ESS2 compensates for the power missing. The output power of ESS is obtained according to Eq. (25).

25 $$P_{ESS} = P_{load} - \sum\limits_{i = 1}^{2} {P_{fcsi} }$$

The output power of ESS1 and ESS2 can be computed as:

26 $$\begin{gathered} P_{ESS1} \left( t \right) = \min \left( {P_{ESS} \left( t \right),P_{ESS1}^{\max } } \right),P_{ESS} \ge 0 \hfill \\ P_{ESS1} (t) = \max \left( {P_{ESS} (t), - P_{ESS1}^{\max } } \right),P_{ESS} < 0 \hfill \\ P_{ESS2} \left( t \right) = P_{ESS} \left( t \right) - P_{ESS1} \left( t \right) \hfill \\ \end{gathered}$$

The energy stored in the ESS at time *t* is shown in Eq. (27), and the SOC of the ESS at time *t* can be expressed as Eq. (28)^25^.

27 $$E_{ESSi} \left( t \right) = \left\{ \begin{gathered} E_{ESSi} \left( {t - 1} \right) - \left( {P_{ESSi} \left( {t - 1} \right) \times \eta_{ch} \times \Delta t} \right),P_{ESSi} < 0 \hfill \\ E_{ESSi} \left( {t - 1} \right) - \left( {P_{ESSi} \left( {t - 1} \right)/\eta_{dis} \times \Delta t} \right),P_{ESSi} \ge 0 \hfill \\ \end{gathered} \right.$$

28 $$SOC_{i} \left( t \right) = \frac{{E_{ESSi} \left( t \right)}}{{E_{rate} }}$$

In these equations Δ*t* is the time step, *η*_*ch*_ and *η*_*dis*_ are the charging and discharging efficiency of the ESS, and *E*_*rate*_ is the rated capacity of the ESS.

### Strategy 2: daisy chain strategy

The daisy chain strategy takes the load power, battery SOC, and MP of the FCS as inputs and determines the FCS starts sequence randomly^24^. After the first FCS output power reaches the maximum, the second FCS starts. The FCS output power is shown in Eqs. (29)–(30). The output power of ESS1 and ESS2 is calculated according to Eqs. (25)–(26).

29 $$P_{fcs1} \left( t \right) = \min (P_{load} ,P_{fcs1} \left( {t - 1} \right) + \Delta P_{fcs}^{\max } )$$

30 $$P_{fcs2} \left( t \right) = \left\{ \begin{gathered} 0,P_{fcs1} \le P_{fcs1}^{\max } \hfill \\ P_{fcs2} \left( {t - 1} \right) + \Delta P_{fcs}^{\max } ,P_{fcs1} > P_{fcs1}^{\max } \hfill \\ \end{gathered} \right.$$

where Δ*Pmax fcs* represents the maximum rate of change of FCS output power (4.24 kW/s).

### Strategy 3

**Figure 9:**
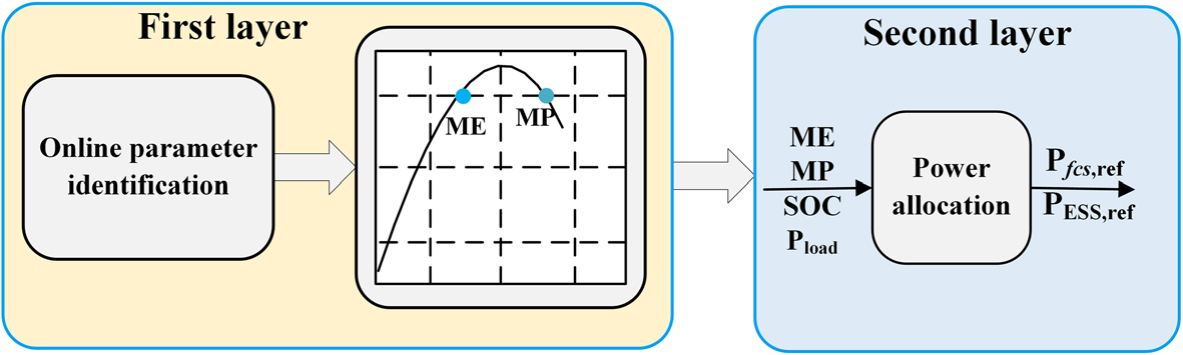
The structure of the proposed power allocation strategy.

The configuration of the proposed power allocation strategy is shown in Fig. 9, which consists of two layers. A fuel cell semi-empirical model combined with AKF is adopted to update the MP and ME of the FCS in the first layer. In the second layer, the performance of the fuel cell is evaluated using Eq. (31), and then the load power is shared among power sources.

31 $$\lambda_{i} \left( t \right) = \frac{{P_{fcsi}^{\max } \left( t \right)}}{{\max (P_{fcs1}^{\max } ,P_{fcs2}^{\max } )}}$$

The FCS output power is shown in Eq. (32). The output power of ESS1 and ESS2 is calculated according to Eqs. (25)–(26).

32 $$\begin{aligned} P_{fcs1} \left( t \right) = & \frac{{\lambda_{1} \left( t \right)}}{{\lambda_{1} \left( t \right) + \lambda_{2} \left( t \right)}} \times P_{load} \left( t \right) \\ P_{fcs2} \left( t \right) = & \frac{{\lambda_{2} \left( t \right)}}{{\lambda_{1} \left( t \right) + \lambda_{2} \left( t \right)}} \times P_{load} \left( t \right) \\ \end{aligned}$$

### Constrains

(1) Due to the slow dynamic characteristics of the FCS, the rate of change of the FCS output power is limited as shown in Eq. (33).

33 $$\left| {P_{fcs} (t + 1) - P_{fcs} (t)} \right| \le \Delta P_{fcs}^{\max }$$

(2) Eq. (34) limits the output power of the FCS and ESS.

34 $$\left| {P_{ESS} } \right| \le P_{ESS}^{\max }$$

(3) To reduce ESS loss, the SOC should keep in the specified range^26^.

35 $$SOC_{\min } \le SOC \le SOC_{\max }$$

## Simulation result

**Figure 10:**
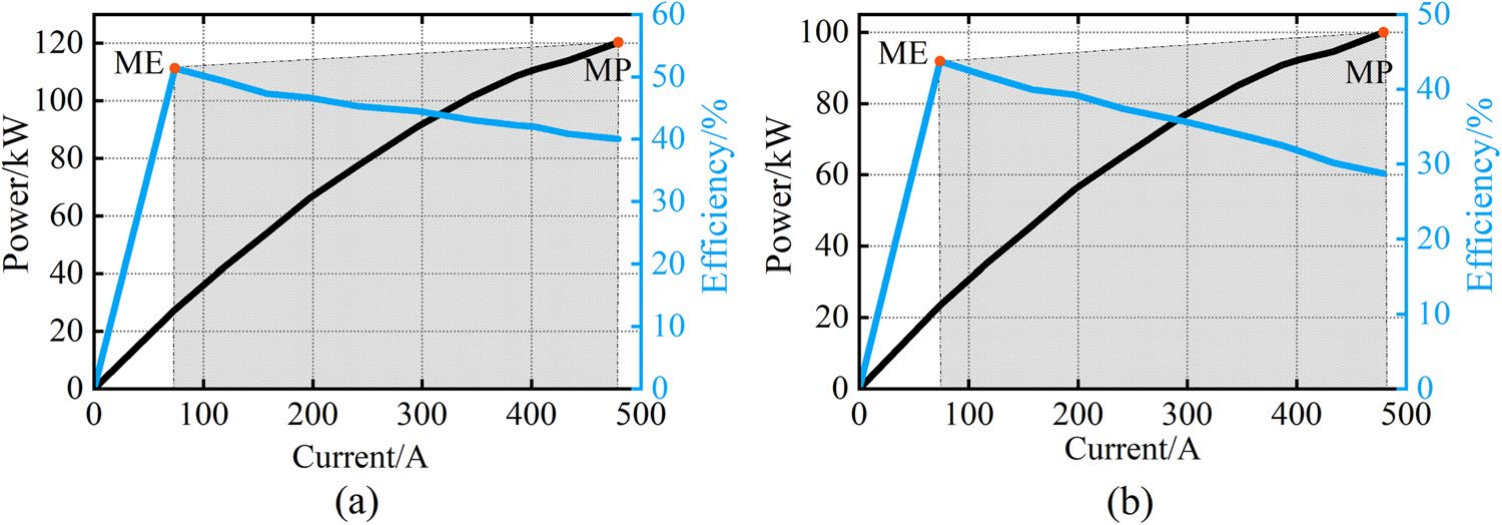
Characteristic curves. (**a**) FCS1; (**b**) FCS2.

This paper takes the hydrogen fuel cell passenger ship as the research object. Two FCSs with different performances are used in this paper. The characteristic curves of FCS1 and FCS2 are shown in Fig. 10. The parameters of the FCS and the ESS are shown in Table 3.

**Table 3:** The parameters of the HES.

	Parameter	Value
FCS1	MP (kW)	120
	ME	51.34%
FCS2	MP (kW)	100
	ME	43.72%
ESS	*SOC*_*max*_	0.9
	*SOC*_*min*_	0.3
	*η*_ch_	0.95
	*η*_dis_	0.97
	*Pmax ESS*(kW)	240

The proposed power allocation strategy is verified with two load profiles. The purpose of designing the first scenario is to illustrate how the three strategies reconfigure the start sequence of the FCS according to their performance. In the second scenario, the load profile of the cruise ship 'Alsterwasser' is used to calculate the total output power of FCS and the hydrogen consumption.

### Scenario 1

**Figure 11:**
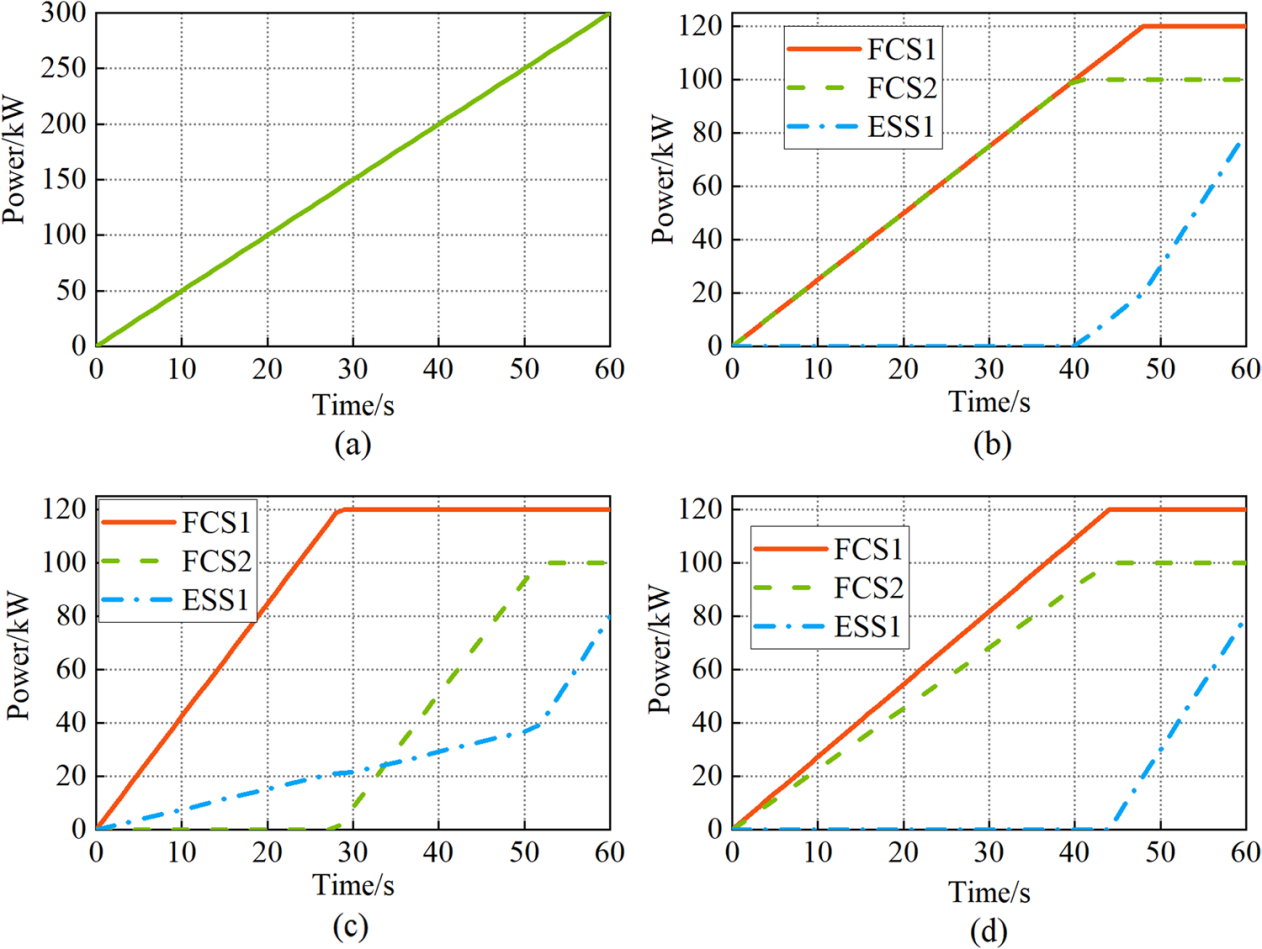
(**a**) Ramp power; (**b**) Strategy 1; (**c**) Strategy 2; (**d**) Strategy 3.

The ramp power is used in the first scenario, as shown in Fig. 11a, the load power increased from 0 to 300 kW.

Figure 11b is the result of the equal allocation strategy. The FCS1 and FCS2 output power rise at the same rate within 0–40 s. The FCS2 output power reaches the maximum at the 40 s. The FCS1 output power reaches the maximum at 48 s. The FCS output power keeps constant after reaching the maximum. The ESS1 is started at 41 s.

Figure 11c is the result of the daisy chain strategy. The start sequence of FCS1 and FCS2 is random. So assuming FCS1 starts first. FCS2 does not start within 0-28 s. FCS1 and ESS1 supply power to the load. The FCS1 output power reaches the maximum at 29 s, and the FCS2 starts. The FCS2 output power reaches the maximum at 52 s. The ESS1 output power is always greater than zero during the operation.

Figure 11d is the result of the proposed power allocation strategy. FCS1 and FCS2 start at the same time, but FCS1 output power is always higher than FCS2, which means FCS1 performance is better. The FCS1 and FCS2 output power reaches the maximum at 44 s and the ESS1 starts.

Hydrogen consumption consists of two parts. The first part is the hydrogen consumed (HC1) in the course of ship operation, and the second part is the hydrogen consumed (HC2) to recharge the ESS1 to its initial charge after the voyage. As shown in Table 4.

**Table 4:** Hydrogen consumption and ESS charge.

		HC1(g)	SOC (%)	HC2(g)	Total(g)
Strategy 1		188.7	79.83	14.8	203.5
Strategy 2	FCS1 starts first	176.3	79.64	31.1	207.4
	FCS2 starts first	198.1	79.8	17.6	215.7
Strategy 3		187.3	79.84	13.9	201.2

The hydrogen consumption is 203.5 g and the final SOC of ESS1 is 79.83% with the equal allocation strategy.

The hydrogen consumption is 207.4 g and the final SOC of ESS1 is 79.64% with the daisy chain strategy when the FCS1 starts first. Compared with the equal allocation strategy, the final SOC of ESS1 reduces by 0.19%, and the HC2 increases by 16.3 g. When the FCS2 starts first, the HC1 increases by 9.4 g compared to the equal allocation strategy due to the poor performance and low efficiency of the FCS2.

The hydrogen consumption is 201.2 g and the final SOC of ESS1 is 79.84% with the proposed power allocation strategy. Among the three strategies, the final SOC of ESS1 is the highest and the total hydrogen consumption is the lowest.

### Scenario 2

**Figure 12:**
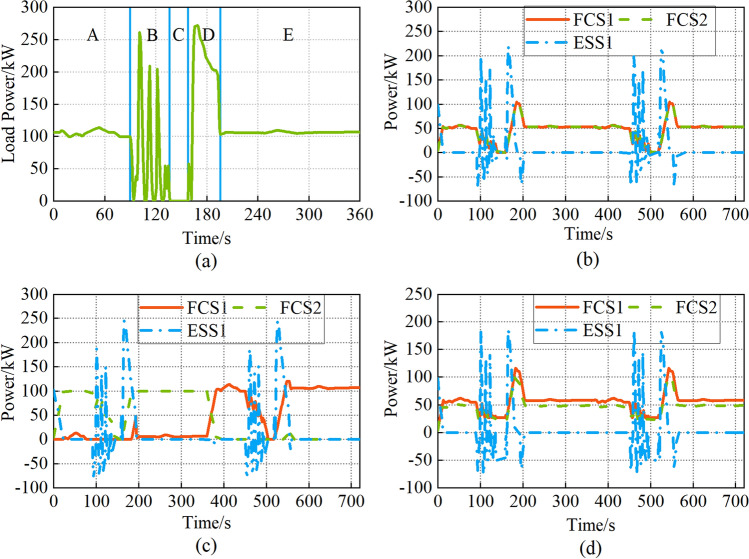
(**a**) Load power; (**b**) Strategy 1; (**c**) Strategy 2; (**d**) Strategy 3.

The load profile of the hydrogen fuel cell passenger ship is shown in Fig. 12a, and the ship operation is divided into four phases, namely, cruising, docking, anchoring, and sailing^5^. Assume the ship needs two repetitions of such a drive cycle.

Figure 12b is the result of the equal allocation strategy. The FCS1 and FCS2 output power is the same due to the fuel cell performance difference are not considered. In the initial stage, the ESS1 supplies power to the load due to the poor dynamic performance of the FCSs. The peak output power of the ESS1 is 219 kW.

Figure 12c is the result of the daisy chain strategy. The FCS2 starts first within 0-360 s, and the FCS1 starts first within 361–720 s. The peak output power of the ESS1 is 244.4 kW, which is the highest among the three strategies.

Figure 12d is the result of the proposed power allocation strategy. FCS1 and FCS2 start at the same time, but FCS1 output power is always higher than FCS2, which means FCS1 performance is better. The FCS output power is not reduced to 0 at 137–158 s and 497–518 s because the operation range of FCS is constrained by the ME and MP. The FCS operates at the highest efficiency point to recharge the ESS1 during the anchoring phase. The peak output power of the ESS1 is 185.9 kW.

**Figure 13:**
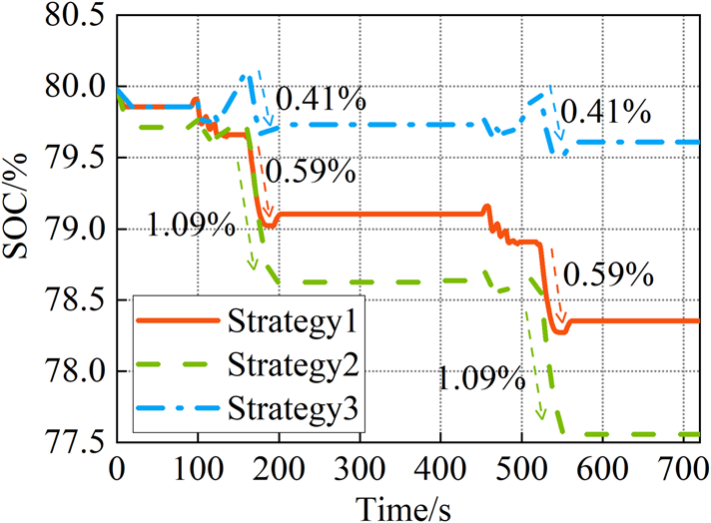
ESS1 charge.

The variation of ESS1 SOC is shown in Fig. 13. The initial SOC of ESS1 is 80%. The maximum depth of discharge (DOD) is 1.09% and the final SOC of ESS1 is 77.58% with the daisy chain strategy.

The maximum DOD is 0.59% and the final SOC of ESS1 is 78.35% with the equal allocation strategy.

The peak output power of the ESS1 is lowest at the sailing phase with the proposed power allocation strategy, the maximum DOD is 0.41%, and the final SOC of the ESS1 is 79.6%. Compared with the equal allocation strategy and the daisy chain strategy, the maximum DOD is reduced by 30.5% and 62.3%, and the final SOC of the ESS1 is improved by 1.6% and 2.6%.

**Figure 14:**
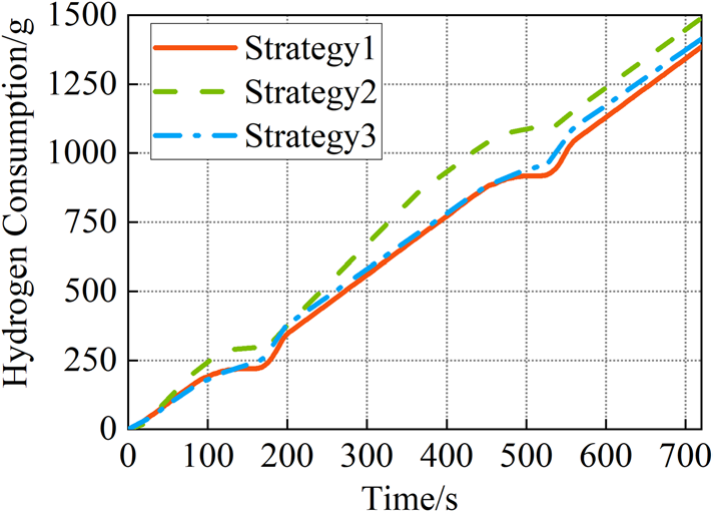
Hydrogen consumption during operation.

The HC1 and HC2 are shown in Fig. 14 and Table 5. The FCS output power is lower with the equal allocation strategy, so the HC1 is less. The total hydrogen consumption is 1531 g. The total output power of FCS2 is 40,995.7 kW.

**Table 5:** Total hydrogen consumption and final SOC of ESS1.

	HC1(g)	SOC (%)	HC2(g)	Total(g)
Strategy 1	1385.1	78.35	145.9	1531
Strategy 2	1489.5	77.56	216.4	1705.9
Strategy 3	1414.3	79.61	34.7	1449

The efficiency is reduced due to the high FCS output power with the daisy chain strategy, and the HC1 is the maximum among the three strategies. The total hydrogen consumption is 1705.9 g. The total output power of FCS2 is 41,759.7 kW.

The HC1 is not the lowest since the operation range of FCSs is limited with the proposed power allocation strategy, the FCSs operate at the highest efficiency point to recharge the ESS1 during the anchoring phase. Therefore, the SOC of ESS1 is the highest, and the HC2 reduces significantly. The total hydrogen consumption is 1449 g. The total output power of FCS2 is 35,207.4 kW. Compared to the equal allocation strategy and the daisy chain strategy, the total hydrogen consumption reduces by 5.3% and 15.1%, and the total output power of FCS2 reduces by 14.1% and 15.7%.

## Conclusion

In this paper, a two-layer power allocation strategy is proposed for a fuel cell ship with two FCSs and two ESSs.

In the first layer, an online parameter identification model consisting of a fuel cell semi-empirical model and AKF is used to continuously update the MP and ME of FCS. Compared with KF and RLS, the MP and ME of FCS obtained by AKF have smaller errors than the real values.

In the second layer, the power allocation is performed according to the performance of the fuel cell. Two load profiles are used to verify the proposed power allocation strategy. The first is used to show how the three strategies reconfigure the start sequence of the FCS. The second is a real load profile, it is used to calculate hydrogen consumption and the total output power of FCS. The total hydrogen consumption is 1449 g and the total output power of the FCS with poor performance is 35,207.4 kW with the proposed power allocation strategy. Compared to the equal allocation strategy and daisy chain strategy, the total hydrogen consumption reduces by 5.3% and 15.1%, and the total output power of the FCS with poor performance reduces by 14.1% and 15.7%.

Collectively, the results show that the proposed method can reduce the operational burden of the FCS with poor performance and improve the efficiency of the system. The results of this paper provide the following directions for future research:

1. The method proposed in this paper is a rule-based power allocation strategy. Future work will develop an optimization-based power allocation strategy.
2. A cost function containing hydrogen consumption and battery energy storage system loss is considered in future research.

## Data Availability

The datasets used and analysed during the current study available from the corresponding author on reasonable request.
